# Understanding the Determinants of BnAb Induction in Acute HCV Infection

**DOI:** 10.3390/v10110659

**Published:** 2018-11-21

**Authors:** Alexander P. Underwood, Melanie R. Walker, Nicholas A. Brasher, Auda A. Eltahla, Lisa Maher, Fabio Luciani, Andrew R. Lloyd, Rowena A. Bull

**Affiliations:** School of Medical Sciences and the Kirby Institute, Faculty of Medicine, UNSW Australia, Sydney, NSW 2052, Australia; a.underwood@unsw.edu.au (A.P.U.); melanie.walker@unsw.edu.au (M.R.W.); nbrasher@kirby.unsw.edu.au (N.A.B.); a.eltahla@unsw.edu.au (A.A.E.); lmaher@kirby.unsw.edu.au (L.M.); luciani@unsw.edu.au (F.L.); a.lloyd@unsw.edu.au (A.R.L.)

**Keywords:** hepatitis C virus, neutralising antibodies, vaccine, reinfection

## Abstract

Despite recent advances in curative therapy, hepatitis C virus (HCV) still remains a global threat. In order to achieve global elimination, a prophylactic vaccine should be considered high priority. Previous immunogens used to induce broad neutralising antibodies (BnAbs) have been met with limited success. To improve immunogen design, factors associated with the early development of BnAbs in natural infection must first be understood. In this study, 43 subjects identified with acute HCV were analysed longitudinally using a panel of heterogeneous HCV pseudoparticles (HCVpp), to understand the emergence of BnAbs. Compared to those infected with a single genotype, early BnAb development was associated with subjects co-infected with at least 2 HCV subtypes during acute infection. In those that were mono-infected, BnAbs were seen to emerge with increasing viral persistence. If subjects acquired a secondary infection, nAb breadth was seen to boost upon viral re-exposure. Importantly, this data highlights the potential for multivalent and prime-boost vaccine strategies to induce BnAbs against HCV in humans. However, the data also indicate that the infecting genotype may influence the development of BnAbs. Therefore, the choice of antigen will need to be carefully considered in future vaccine trials.

## 1. Introduction

Despite recent advances in curative therapy, hepatitis C virus (HCV) still remains a global threat. Of the 70 million people chronically infected with HCV worldwide, by 2015, only 13% had received curative treatment and only 20% had been diagnosed [1,2]. If left untreated, chronic HCV infection leads to progressive hepatic fibrosis, culminating in cirrhosis, liver failure, and an increased risk of hepatocellular carcinoma [3]. As transmission of HCV is parenteral, it is most common amongst people who inject drugs [4,5,6]. In most cases, acute HCV infection remains asymptomatic, allowing transmission of the virus to go undetected. Reinfection in this high-risk population is significant, and presents a significant challenge for elimination strategies [7,8]. For these reasons, a prophylactic vaccine still remains a priority as curative therapy alone is insufficient for global elimination [2].

To date, most prophylactic vaccines confer protection via neutralising antibodies (nAbs). In HCV, increasing evidence has shown that the emergence of nAbs, which target the envelope glycoproteins E1 and E2, play a crucial role in viral control in acute infection [9,10,11,12,13]. However, reinfection in high-risk populations suggests that the development of protective nAbs is difficult [9,10,14,15,16]. This may be due to the vast heterogeneity of HCV across populations, represented by 7 major genotypes (Gt) and over 60 subtypes [17]. In addition, the error-prone replication machinery results in rapidly mutating progeny, termed quasispecies, capable of evading host immune responses [18,19]. It is proposed that broad neutralising antibodies (BnAbs), capable of neutralising multiple genotypes, will need to be induced if a vaccine is to be protective [20,21]. So far, evidence from passive immunisation studies in animal models has suggested that BnAbs are capable of protection against a clonal viral inoculum [14,15,22]. However, passive immunisation in humans has only managed to delay infection of the donor liver following transplantation of HCV-infected patients, suggesting that protection via BnAbs alone remains distant [23].

Previous attempts to induce BnAbs in humans and animal models using recombinant immunogens has been met with limited success [23,24]. Antibodies isolated from these subjects, while neutralising, were isolate-specific, implying that better immunogens are required. Other immunisation strategies, including multivalent and prime-boost immunisations, have been met with better success by inducing BnAbs in animal models with a breadth comparable to that in chronic infection [25,26]. However, the efficacy of these immunisation strategies, in humans, is yet to be determined.

Currently, little is known about the virological factors associated with successful induction of BnAbs in natural HCV infection. Studies in human immunodeficiency virus (HIV) have revealed the emergence of BnAbs to be associated with several parameters, including viral load, viral genotype, and duration of infection [27,28,29]. In HCV, BnAbs are often delayed in appearance until chronic infection has been established, and the factors associated with emergence of BnAbs remain to be discovered [11,30,31,32]. Understanding exactly when BnAbs emerge has been challenging, due to the difficulty in detecting the typically asymptomatic, acute HCV infections in marginalised at-risk populations.

In this study, the development of BnAbs in acute HCV infection was assessed in incident cases from a prospective cohort of HCV-negative, high-risk, injecting drug users. The development of BnAbs was assessed against a range of virological parameters, including viral genotype, duration of infection, mixed infection (more than one infecting HCV subtype), and reinfection. In subjects infected with only one HCV subtype (termed here mono-infected) nAbs were not seen to broaden until a late persistent infection had been established. In contrast, subjects co-infected with at least two HCV subtypes (incident mixed) developed broad nAb responses during early acute infection. Subjects who cleared, and then became reinfected, only demonstrated broader nAb responses upon re-exposure to HCV. Collectively, the findings indicate that BnAbs are rarely elicited in a timely fashion in natural HCV infection.

## 2. Materials and Methods

### 2.1. Study Cohort

The Hepatitis C Incidence and Transmission Studies (HITS) were prospective cohorts of high-risk, HCV-seronegative/RNA-negative injecting drug users recruited from the prisons (HITS-p) and the general community (HITS-c) in New South Wales, Australia. A total of 590 subjects from 34 correctional centres were enrolled between 2005 and 2012 in HITS-p, and 268 subjects were recruited from three urban Sydney regions (southwest, inner metropolitan, and western Sydney) between 2008 and 2014 in HITS-c. Details of these cohorts have been previously reported [33,34,35,36,37,38,39,40,41]. All serum was tested for anti-HCV antibodies using the Abbott ARCHITECT anti-HCV chemiluminescent microparticle immunoassay (CIA) (Abbott Diagnostics, Chicago, IL, USA) and HCV RNA quantification was performed using either the VERSANT HCV RNA Qualitative Transcription Mediated Amplification (TMA) assay (Bayer Diagnostics, Emeryville, CA, USA) or the COBAS AmpliPrep/ COBAS TaqMan HCV assay (Roche, Basel, Switzerland), as previously described [37].

### 2.2. Ethics

Ethical approvals were obtained from the Human Research Ethics Committees of Justice Health (reference number GEN 31/05), the New South Wales Department of Corrective Services (reference number 05/0884), and the University of New South Wales (reference numbers 05094 and 08081), all located in Sydney, Australia. Written informed consent was obtained from the participants.

### 2.3. Anti-Envelope Antibody ELISA

Subject plasma was screened for anti-envelope IgG as previously described [42]. Briefly, Nunc immuno-microtitre plates (Thermo Fisher Scientific, Waltham, MA, USA) were prepared by coating each well with 500 ng of *Galanthus nivalis* lectin (GNA; Sigma-Aldrich, St. Louis, MO, USA), followed by blocking the wells with 5% skim milk (Thermo Fisher Scientific) diluted in TBS-T (20 mM Tris-HCl, pH 7.5, 150 mM NaCl, 0.1% Tween 20) overnight. Lysates of HEK293T cells expressing H77.20 or UKN3A.13.6 E1/E2 were captured on the GNA-coated plates. Subject plasma was heat inactivated and diluted 1:10 in 5% skim milk TBS-T, and added to the bound antigen. Binding of IgG in plasma was detected using mouse anti-human IgG conjugated with horseradish peroxidase (Jackson ImmunoResearch, West Grove, PA, USA), followed by the addition of 3,3’,5,5’-tetramethylbenzidine (TMB; Thermo Fisher). The reaction was stopped after 15 min with 1 M hydrochloric acid, and plates were read at 450 nm on a CLARIOstar microplate reader (BMG Labtech, Mornington, Victoria, Australia). The Ab titre was expressed as a ratio of raw OD from test plasma samples and the OD of a healthy plasma sample (calculated as OD_test plasma_/OD_healthy plasma_). The ratio represents the fold difference from the healthy control.

### 2.4. HCVpp Production and Neutralisation Assay

All E1/E2 expression plasmids were previously tested in the HCVpp system (kindly gifted by Jonathan Ball) [43]. A total of 11 HCV E1/E2 expression plasmids, representing the 6 major HCV genotypes, were used to generate heterologous HCVpp. This included H77.20 (GenBank accession AF011751), UKN1A20.8 (EU155192), UKN1B5.23 (AY734976), UKN2A1.2 (AY734977), UKN2B2.8 (AY734983), UKN3A1.28 (AY734984), UKN3A1.9 (AY734985), UKN3A13.6 (AY894683), UKN4.11.1 (AY734986), UKN5.14.4 (AY785283), and UKN6.5.340 (AY736194). The luciferase-encoding reporter plasmid (pTG126), and the murine leukaemia virus (MLV) *gag/pol*-encoding packaging construct (phCMV-5349) were kindly provided by Prof. Francois-Loic Cosset (University of Lyon, France).

Retroviral HCV pseudoparticles (HCVpps) were prepared as described elsewhere [43,44], based on the protocols developed by Bartosch and colleagues [45]. In brief, HCVpp was generated by co-transfecting pTG126, phCMV-5349, and a HCV E1/E2 clone onto Lenti-XR HEK293T cells (Takara, Mountain View, CA, USA), seeded the night before using a mammalian Calphos transfection kit (Takara Bio, Mountain View, CA, USA). The infectivity of the HCVpp was titrated to standardise all HCVpp to be 5–20-fold more infectious than mock pseudoparticle lacking any HCV E1/E2. Neutralisation assays were performed as previously described [9,46]. In brief, titrated HCVpp was incubated for 1 h with heat-inactivated plasma at a 1:50 dilution, before being added to Huh7.5 cells (Apath, New York, NY, USA). After 72 h, the cells were lysed with a lysis buffer (Promega, Madison, WI, USA), Bright Glo reagent (Promega) was added, and luminescence was measured on a CLARIOstar microplate reader (BMG Labtech). Percentage neutralisation was calculated as (1 − RLU_test plasma_/RLU_healthy control_) × 100. In addition to HCVpp, VSV-g pseudoparticles were tested against all subject’s plasma, to demonstrate the specificity of nAbs.

### 2.5. Measuring Breadth of nAbs in Plasma

To assess the breadth of nAb responses in plasma, a neutralisation score adapted from a HIV study was applied [28]. The scoring system accounted for both the breadth and potency of neutralising responses in plasma, whereby >80% neutralisation received a score of 3, 50%–80% neutralisation received a score of 2, 20%–49% neutralisation received a score of 1, and <20% neutralisation received a score of 0. Each HCVpp was scored individually, meaning that the maximum cumulative score for each plasma sample was 33.

### 2.6. Statistical Tests

Statistical tests were performed using GraphPad Prism software (version 7, LaJolla, CA, USA). All unpaired data was analysed using the Mann–Whitney *t*-tests, and all paired data was analysed using the Wilcoxon *t*-test. Statistical significance was defined as a *p* value less than 0.05. Results were expressed as a mean ± standard deviation.

## 3. Results

### 3.1. Assessing the Breadth of nAbs in HCV Infection

Of the 828 subjects enrolled in the HITS-p and HITS-c cohorts, 342 (41.3%) became positive for HCV infection, identified either through serological testing or RNA positivity. Of these 342 subjects, 126 (36.8%) were identified with acute infection, and had pre-infection samples allowing calculation of the estimated days post-infection (DPI) for each follow-up sample, as described previously [47]. Of these, 9 (7.1%) were identified as incident-mixed infection (co-infected with more than one HCV subtype at the first available viraemic time point). These subjects, as well as 34 mono-infected subjects with adequate longitudinal follow up, were selected for this study. Of these 43 subjects, 16 (37.2%) had subsequent documented episodes of reinfection. A summary of patient demographic and virological data can be found in Table 1.

**Figure 1:**
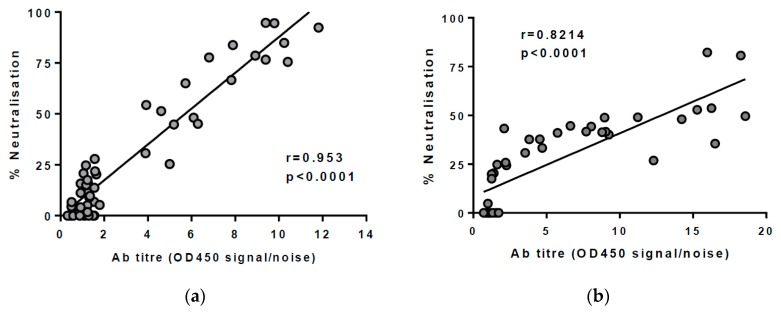
Linear regression analysis of anti-E1/E2 IgG titres (optical density 450 signal/noise) and percentage neutralisation (%) of HCVpp carrying the same envelope sequence. A significant correlation was found in (**a**) matched Gt1 envelope sequences (H77.20, *r* = 0.953, *p* < 0.0001) and (**b**) matched Gt3 envelope sequences (UKN3A.13.6, *r* = 0.8214, *p* < 0.0001) demonstrating that neutralisation of HCVpp was likely be associated with anti-envelope IgG in the plasma.

All subjects were initially screened for anti-envelope antibodies using an HCV-specific IgG detection ELISA. To confirm that neutralisation in plasma was due to HCV-specific IgG responses, the anti-envelope antibody titre (OD_450_ signal/noise) was compared to the neutralisation of a HCVpp carrying the reference envelope sequence (H77.20 or UKN3A13.6). As shown in Figure 1a,b, a significant correlation was found (*r* = 0.953, *p* < 0.0001; and *r* = 0.821, *p* < 0.0001), indicating that neutralisation of HCVpp was likely to be due to the presence of anti-envelope IgG.

**Figure 2:**
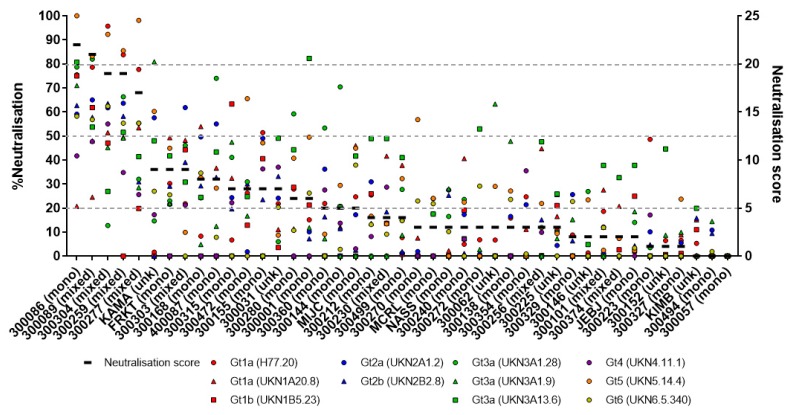
Individual analysis of the neutralisation activity against 11 HCVpp for the 43 subjects included in this study. Subject ID is indicated on the x axis, and infection history (mono-, mixed, or unknown [unk] infection, is indicated in parentheses). The neutralisation score (black line for each subject and right-hand y axis) applied for each subject (listed on the x axis) is observed to summarise both the breadth (multiple HCVpp neutralised) and neutralisation potency (left-hand y axis) of each HCVpp, representing an accurate summary of overall neutralisation potential. Each grey line represents the cut off for each part of the scoring system.

**Figure 3:**
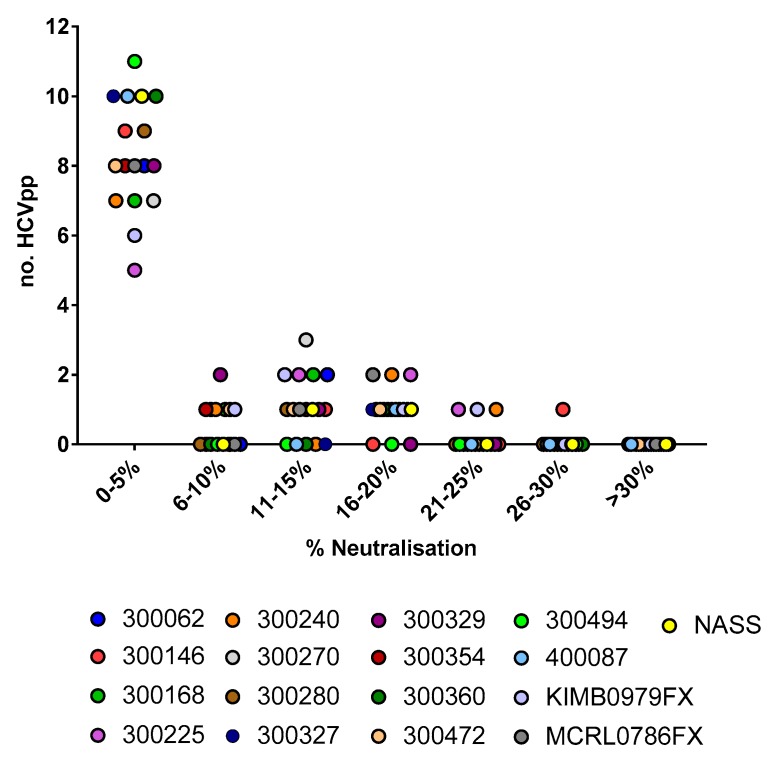
Neutralising activity of available plasma samples (diluted 1:50) collected pre-HCV infection (*n* = 17) compared to a healthy control. All 17 subjects were tested against a panel of 11 HCVpp, representing GT1 to 6. While all subjects demonstrated some neutralising activity, this was below the 20% cut off (grey line) with the exception of single HCVpp for subjects 300146 (UKN3A1.9, 27.9% neutralisation), 300225 (UKN1A20.8, 21.0% neutralisation), KIMB0979FX (UKN3A13.6, 21.7% neutralisation), and NASS0384MX (UKN3A1.9, 20.5% neutralisation). Given that 97.9% of neutralising activity was below this threshold, this validated the use of a lower neutralisation cut off.

To assess the breadth of neutralisation in plasma, a panel of 11 HCVpp, representing envelope proteins from HCV genotypes 1–6, was used. As shown in Figure 2, neutralisation breadth and potency against these HCVpp was summarised using a neutralisation score adapted from a comparable approach taken in HIV [28]. Given that it is unclear what percentage of neutralisation has biological relevance in vivo, this approach avoided the application of a single arbitrary cut off. However, to assess the validity of a lower cut off (20% neutralisation), 17 subjects that had plasma available pre-infection (*n* = 17) were compared to a healthy control. As shown in Figure 3, 14 of the 17 subjects’ neutralising activity against the 11 HCVpp were all under the 20% cut off. The three remaining subjects each reacted to only 1 of the 11 HCVpp tested: subject 300146 (UKN3A1.9, 27.9% neutralisation), 300225 (UKN1A20.8, 21.0% neutralisation), KIMB0979FX (UKN3A13.6, 21.7% neutralization), and NASS0384MX (UKN3A1.9, 20.5% neutralisation). Given that 97.9% (4 of 187 HCVpp) of the neutralising activity was below this threshold, this validated the use of a lower neutralisation cut off.

### 3.2. Early Induction of Broad nAbs Was Associated with Incident Mixed Infections

**Figure 4:**
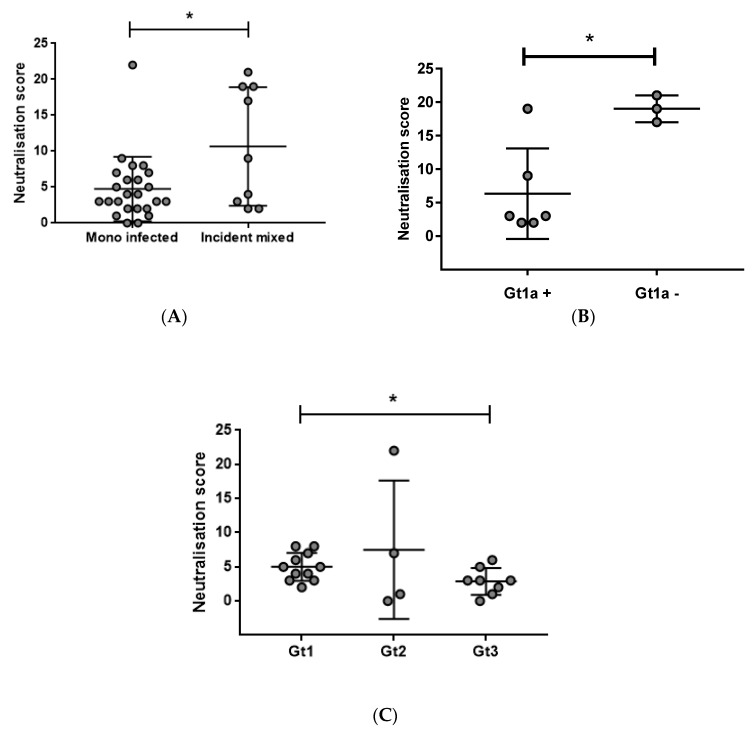
Comparison of neutralisation scores in different genotype infections. (**A**) Incident mixed subjects had a significantly greater neutralisation score compared to those who were mono-infected (*p* = 0.0123, Mann–Whitney *t*-test). (**B**) Incident mixed subjects that did not have Gt1a detected during infection had a significantly higher neutralisation score than those that did (*p* = 0.0495, Mann–Whitney *t*-test). (**C**) In mono-infections, those infected with Gt1 had a significantly higher neutralisation score compared to Gt3 mono-infections (*p* = 0.0458, Mann–Whitney *t*-test). Subjects 300138, 300144, and 300155 were excluded from this analysis due to their first viraemic time points being estimated to have been detected after chronic viraemia had been established.

Upon ranking the neutralisation scores (Figure 2), it was noted that many of the highest-ranked subjects had an incident mixed infection. To evaluate whether harbouring two genotypes, compared to a single genotype, influenced the development of nAb breadth and potency, neutralisation scores between incident mixed (*n* = 9) and mono-infected (*n* = 31) subjects were compared (Figure 4A). Subjects 300138, 300144, and 300155 were excluded from this analysis due to their first viraemic time point being estimated to have been detected during the chronic stages of infection (>180 DPI). Incident mixed subjects were found to have significantly higher neutralisation scores (*p* = 0.0126) suggesting that the presence of multiple infecting genotypes influences early BnAb development. However, neutralisation scores between incident mixed subjects were also noted to vary considerably (Figure 4A). To address whether this was related to particular combinations of genotypes, incident mixed subjects were separated based on the infecting genotypes, and the neutralisation scores were compared (Figure 4B). Despite the limited sample size, subjects infected with a non-Gt1a strain had a significantly higher neutralisation score compared to those with Gt1a (*p* = 0.0495), suggesting that the infecting genotype may influence BnAb development. Further, as shown in Figure 4C, those mono-infected with Gt1 had a moderate, but significantly broader nAb response, than those infected with Gt3 (*p* = 0.0458). It should be noted that a single outlier subject infected with Gt2 had the best overall neutralisation score in the mono-infection group (subject ID: 300086), however, this was not found in others infected with the same genotype (Figure 4C). Collectively, this data indicates that the BnAb responses during acute HCV infection may be associated with the presence of multiple genotypes, particularly multiple genotypes with the exclusion of Gt1a.

### 3.3. Breadth of nAbs is Boosted with Re-Exposure to HCV

**Figure 5:**
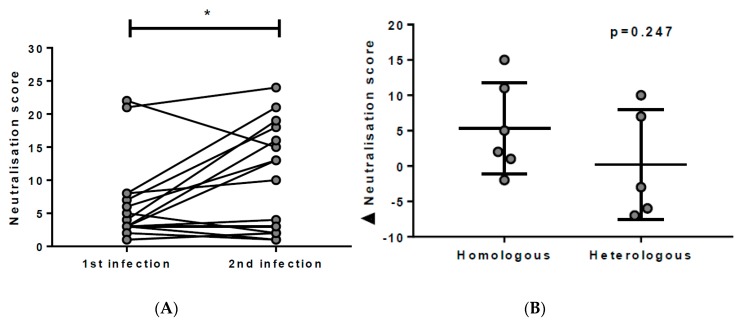
Comparison of neutralisation scores between primary and secondary infections. (**A**) Neutralisation scores in acute secondary infections were found to be significantly higher than acute primary infections (*p* = 0.0494, Wilcoxon *t*-test). (**B**) The difference in neutralisation scores between primary and secondary infections in homologous and heterologous reinfections was not found to be significant (*p* = 0.247, Mann–Whitney *t*-test). Subject 300089 was excluded from this analysis, due to incident mixed primary infection.

In order to assess whether re-exposure to HCV influenced neutralisation breadth, the scores between primary and acute secondary infections were compared in 16 subjects with documented reinfection (Table 1). Collectively, secondary infections had significantly higher neutralisation scores (*p* = 0.0458) than primary infections, suggesting the breadth of nAbs may be boosted upon HCV re-exposure (Figure 5A). In 4 subjects, however, neutralisation scores were not found to be boosted upon re-exposure. Of these 4 subjects, 3 were re-exposed to a heterologous genotype, suggesting that homologous re-exposure may be more beneficial for inducing BnAbs. To further investigate this possibility, neutralisation scores were compared between primary and secondary infections, with homologous and heterologous genotypes upon re-exposure (Figure 5B). Four subjects could not be included in this analysis, due to the viral load being too low to identify the infecting genotype (subject ID: 300062, 300152, 300225, and KAMA0984MX). Generally, those re-exposed to a homologous genotype demonstrated a better boost in neutralisation score, however, this was not significant (*p* = 0.247, Figure 5B).

### 3.4. Prolonged Antigen Exposure Is Associated with Broader nAb Development in Mono-Infection

**Figure 6:**
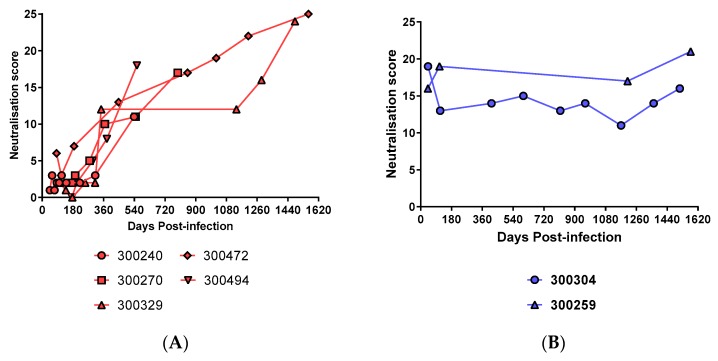
Neutralisation scores analysed longitudinally in persistent (**A**) mono-infections and (**B**) incident mixed infections. Neutralisation scores in mono-infections increased with the duration of the infection. These scores did not reach a comparable level to incident mixed infections until 700 days post-infection. In incident mixed infections, neutralisation scores developed earlier than mono-infections but did not increase with persistent viraemia.

In order to assess the effect of prolonged antigen exposure on nAb responses, neutralisation scores were analysed longitudinally in those that developed a persistent mono-infection (*n* = 6, subject ID: 300240, 300270, 300327, 300329, 300472, and 300494). A linear relationship between duration of infection and neutralisation scores was observed, indicating that nAbs broadened with increased antigen exposure (Figure 6A). However, the time taken for nAbs to broaden was slow, taking years to reach levels similar to those observed for acute incident mixed infections. As two of the incident mixed infection subjects were found to develop a persistent infection (subject ID: 300259 and 300304), these were examined longitudinally, to assess the combined influence of incident mixed and persistent infection (Figure 6B). In contrast to mono-infections, neutralisation scores were not seen to increase with persistent viraemia in incident mixed infections (Figure 6B).

### 3.5. Breadth Is Not Associated with Viral Load

**Figure 7:**
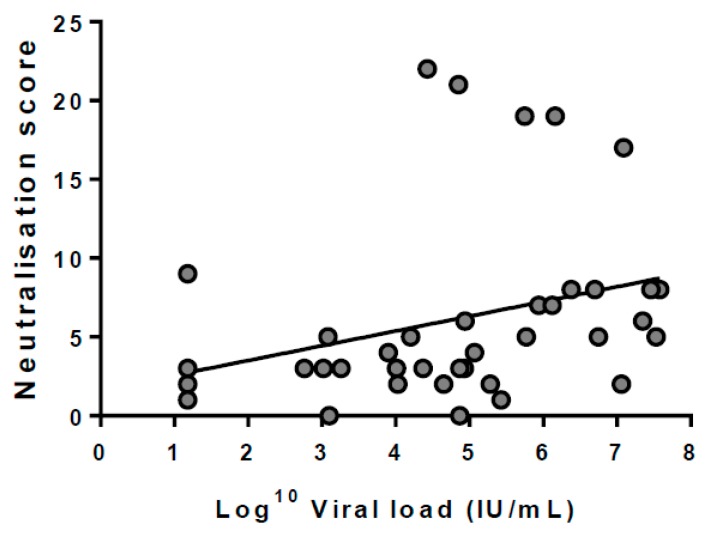
Linear regression analysis between the neutralisation score and peak viral load detected during acute viraemia (IU/mL). Although a modest correlation was found (*r* = 0.299), this was not significant (*p* = 0.0641).

To determine if viral load influenced the emergence of BnAbs, the peak viral load detected during acute infection was compared to the peak neutralisation score. While there was a moderate positive relationship between these variables (*r* = 0.299), this was not found to be significant (*p* = 0.0641, Figure 7).

## 4. Discussion

For highly diverse viruses, such as HCV, a vaccine that can induce BnAbs is considered ideal (reviewed in [20,21]). However, in the context of infection, BnAbs typically do not develop until chronic infection has been established [11,30,31,32], suggesting an intrinsic or virus-induced defect, and, thus, presenting a challenge for vaccine design. This uncertainty highlights the need to better understand the parameters involved in successful induction of BnAbs to inform vaccine development. The present study firstly suggests that breadth in the immunogen (or, in this case, divergent infecting genotypes) represents one such parameter for induction of BnAb responses. Secondly, the data reported here indicate that BnAb responses develop slowly, either reflecting the need for considerable somatic hypermutation, or T cell help, or both. Thirdly, the findings in reinfection generally suggested that memory responses had been established, as boosting was evident upon re-exposure.

By examining antibody responses in a group of acutely infected individuals, the findings presented here indicate that BnAb responses may be best elicited by the presence of multiple immunogens during early HCV infection. Successful vaccines against many pathogens, such as poliovirus and human papilloma virus (HPV), offer their success to polyvalent immunogens, designed to increase the breadth of immune responses by the incorporation of immunogens from multiple strains. In a recent report, mice and macaque animal models immunised against HCV developed broader nAbs when a multivalent vaccine approach was administered [25]. By incorporating more than one immunogen, B cell clones with higher affinity to the variants, either individually or combined, may have a competitive advantage and, thus, broader nAb responses may be elicited. Noting the counter-concern of antigenic competition abrogating BnAb generation, it is reassuring to note that the results presented here, from subjects with incident mixed HCV infection, highlight the potential for a multivalent vaccine approach to induce BnAbs in humans.

The major goal of vaccination is to generate long-term protection against infection. For pathogens like hepatitis B virus (HBV), protection is conferred by repeated dose regimens that are designed to generate long-lasting immunity. In previous reports, episodes of reinfection have been reported to boost antibody titres against HCV, however, the impact of these exposures on breadth were not investigated [9,10]. Building upon these studies, the limited dataset presented in this study indicates that neutralising responses generally broadened when re-exposed to HCV. This outcome has also been observed in animal models undergoing HCV prime-boost vaccine trials [26]. While it is suggested that boosting with a heterologous virus can induce broader immune responses than homologous re-exposure (reviewed in [48]), in the reinfection cases reported here, there were no significant differences between naturally occurring homologues of these immunogens in the form of divergent genotype (heterologous) versus same-genotype (homologous) natural HCV infections.

In this regard, it is important to consider that the E1/E2 proteins generally differ between genotypes by approximately 30%, and between subtypes by approximately 15% (reviewed in [21]). Thus, the within-genotype re-exposure still offers a moderately non-conserved protein. This lack of conservation is generally reflected in hypervariable regions and neutralising antibody domains (reviewed in [20]), which may account for the boost in breadth with homologous re-exposure. It is also worth noting that the prime-boost immunisation strategy relies on B cell responses from the previous exposure being recalled to undergo further affinity maturation in the germinal centre, and usually increased breadth [49]. However, this strategy relies on good memory B cell responses being induced with the primary exposure, and sufficient cross-reactivity with the second immunogen to ensure cross-recognition and, thus, recall of these memory B cell responses. For the minority of subjects that failed to boost upon re-exposure to HCV, this may be reflected by poorly induced memory B cells during the first encounter with the virus (neutralisation score <5). To date, there has been limited investigation of memory recall in HCV. Collectively, the natural history infection data in this study suggests that, in some instances, a prime-boost vaccine regimen can effectively induce BnAbs against HCV.

For other highly diverse RNA viruses, like HIV and influenza, the strain of antigen presented has been shown to significantly impact the breadth of the host immune response [28,50]. The choice of immunogen must be carefully considered if a protective vaccine against HCV is to be developed. In most HCV vaccine trials to date, the formulations used to generate nAbs have typically included Gt1-based envelopes [24,26,51]. However, the present findings indicate that, while Gt1 mono-infections were found to induce broader nAb responses, the presence of Gt1a hindered breadth development in incident mixed infections. The conclusions drawn here are, however, limited by the sample size and the fact that the Gt1 HCVpp may be more closely related to autologous virus compared to the Gt3 HCVpp. Compared to Gt3, Gt1 E1/E2 has a greater intra-host diversity within the quasispecies [52], which may positively influence BnAb development in mono-infections. However, this diversity could dominate antigen presentation in incident mixed individuals masking presentation of antigen from other circulating genotypes. It is, therefore, important that future studies investigate different combinations of HCV antigens, in order to optimise early BnAb induction. While the emergence of nAbs has been linked to improved viral control [12], how or when nAbs begin to broaden remains relatively unknown. Previous studies have linked the early appearance of BnAbs to spontaneous resolution of the virus [11,53]. Previous longitudinal analyses on persistently infected individuals has shown that nAbs begin to become broad around 100 weeks post-infection (700 DPI) [30]. However, the panel of HCVpp used in these studies was limited to only a single genotype (Gt1), and does not accurately reflect cross-genotypic breadth. Building on this, the current data indicate that nAbs continue to broaden slowly with an increasing duration of viral persistence, similar to that seen in HIV infection [54]. Interestingly, the breadth of the nAb response in mono-infections was not comparable to that of broad incident mixed subjects until 700 days of infection had passed. By contrast, BnAbs were induced during early incident mixed infections, but were not influenced by increasing viral persistence. This finding suggests either that the breadth of neutralisation potential in certain individuals is limited or, alternatively, that the delay is a virus-induced phenomenon. Importantly, these data highlight the best means to induce BnAbs early during antigen encounter.

Several cross-sectional studies in HIV have demonstrated a link between the amount of antigen (viral load) and the breadth of nAb responses [55,56]. Interestingly, in chronic HCV, broader antibody profiles have been associated with lower viral loads [57]. However, this latter observation may be due to antibody responses suppressing the viral load in these individuals prior to the subsequent viral escape at the nAb epitope(s) and rise in viral load. Such viral load analyses are often heavily biased by the time at which samples are collected (reflecting the duration of viraemia). Despite attempting to reduce this bias by using the peak viral load detected during acute viraemia, only a modest relationship between viral load and nAb breadth could be determined.

In conclusion, this study shows that the development of broad nAb responses against HCV is difficult, typically taking years to manifest in typical mono-infections. By contrast, the rapid induction of BnAbs in incident mixed infection episodes helps provide the proof-of-principle for multivalent and prime-boost immunisation strategies. The success of these immunisations may rely on the use of carefully selected immunogens with the greatest propensity for induction of BnAbs across individuals.

## Figures and Tables

**Table 1:** Patient data summary.

Subject ID	Gender	Age	Estimated Days Since First Viraemic Time Point	Infecting Subtype ^a^	Peak Viral Load (IU/mL)	Time at Peak Viral Load (DPI) ^b^	Persistent Infection	Reinfecting Subtype	Estimated Days Since First Infection
300001	F	26	155	3a	86,443	155	-	1a	448
300031	M	22	51	Unk	861,000	51	-	-	-
300057	M	22	51	3a	1254	51	-	-	-
300062	F	25	95	Unk	<15	95	-	1b	1079
300086	M	22	51	2a	26,953	51	-	3a	820
300089	M	23	179	1b/3a	70,737	179	-	3a	866
300101	M	23	179	1a/3a	11,441,811	179	-	-	-
300138	M	21	191	3a	582	191	-	3a	386
300144	F	20	268	3a	1205	268	-	1a	630
300146	M	18	170	Unk	<15	170	-	-	-
300152	M	19	122	Unk	<15	122	-	1a	1157
300155	M	27	217	3a	615	217	-	Unk	1468
300168	M	18	4	1b	10,989,916	32	-	1a	120
300212	M	19	146	1b	8039	146	-	1a	358
300223	F	26	169	3a	267,750	169	Y	-	-
300225	M	22	103	Unk	<15	103	-	1b	416
300230	F	26	165	1a/2a	2450	165	-	-	-
300240	M	18	45	3a	54,887	58	Y	-	-
300256	M	24	44	1a/3a	34,149,824	44	Y	-	-
300259	M	20	41	3a/2a	567,122	41	Y	-	-
300270	M	25	173	6a	10,671	173	Y	-	-
300272	F	29	61	3a	10,122	61	-	1a	2270
300277	M	18	39	3a/3b	5,482,503	63	-	-	-
300280	F	19	176	1a	22,384,668	176	-	-	-
300303	F	40	48	1a/3a	5,052,079	48	-	-	-
300304	M	46	43	1a/3a	1,436,063	114	Y	-	-
300315	M	21	51	2b	872,728	51	Y	-	-
300327	M	28	101	2b	6748	146	Y	-	-
300329	M	19	104	3a	9504	177	Y	-	-
300354	M	18	127	1a	23,573	127	-	1a	336
300360	M	23	30	3a	5,648,631	30	-	-	-
300374	M	23	145	1a/3a	44,836	145	-	-	-
300472	M	22	84	1a	1,263,375	84	Y	-	-
300494	F	23	174	2b	73,841	174	Y	-	-
300499	M	23	169	1a	591,430	169	-	-	-
400087	M	23	31	1b	13,118,082	42	-	3a	49
FRKT0686FX	M	22	41	1a	2,404,901	41	-	-	-
JEBJ0991MX	M	18	74	1a	1037	74	-	-	-
KAMA0984MX	M	24	88	Unk	<15	88	-	3a	591
KIMB0979FX	F	29	98	Unk	<15	98	-	-	-
MCRL0786FX	F	22	81	1a	1846	81	-	-	-
MIJC1076FX	F	32	126	1a	15,997	175	-	-	-
NASS0384MX	M	25	84	3a	5321	94	-	3a	84

^a^ Viral genotypes that could not be identified via core, E1/HVR1, or 5′ UTR PCR, as described previously [37], are abbreviated as unknown (Unk). ^b^ Time point used for neutralisation assays.
